# The Role of Species Traits in Mediating Functional Recovery during Matrix Restoration

**DOI:** 10.1371/journal.pone.0115385

**Published:** 2014-12-12

**Authors:** Andrew D. Barnes, Rowan M. Emberson, Frank-Thorsten Krell, Raphael K. Didham

**Affiliations:** 1 School of Biological Sciences, University of Canterbury, Private Bag 4800, Christchurch, New Zealand; 2 Systemic Conservation Biology, Johann-Friedrich-Blumenbach Institute for Zoology and Anthropology, University of Göttingen, Göttingen, Germany; 3 Department of Ecology, Lincoln University, Lincoln, New Zealand; 4 Department of Zoology, Denver Museum of Nature & Science, Denver, Colorado, United States of America; 5 CSIRO Land & Water Flagship, Centre for Environment and Life Sciences, Floreat, Western Australia, Australia; 6 School of Animal Biology, The University of Western Australia, Crawley, Western Australia, Australia; National University of Mongolia, Mongolia

## Abstract

Reversing anthropogenic impacts on habitat structure is frequently successful through restoration, but the mechanisms linking habitat change, community reassembly and recovery of ecosystem functioning remain unknown. We test for the influence of edge effects and matrix habitat restoration on the reassembly of dung beetle communities and consequent recovery of dung removal rates across tropical forest edges. Using path modelling, we disentangle the relative importance of community-weighted trait means and functional trait dispersion from total biomass effects on rates of dung removal. Community trait composition and biomass of dung beetle communities responded divergently to edge effects and matrix habitat restoration, yielding opposing effects on dung removal. However, functional dispersion—used in this study as a measure of niche complementarity—did not explain a significant amount of variation in dung removal rates across habitat edges. Instead, we demonstrate that the path to functional recovery of these altered ecosystems depends on the trait-mean composition of reassembling communities, over and above purely biomass-dependent processes that would be expected under neutral theory. These results suggest that any ability to manage functional recovery of ecosystems during habitat restoration will demand knowledge of species' roles in ecosystem processes.

## Introduction

Significant advances have been made in understanding the cascading effects of global environmental change on biodiversity loss and associated ecosystem functioning [1]–[3]. We have come to understand that the rules governing community assembly provide a strong foundation on which we can interpret the relationship between biodiversity loss, altered species trait composition of communities and declining ecosystem process rates [4]–[6]. Although there has been considerable debate over the relative importance of qualitative (i.e. species identity) versus quantitative (i.e. species richness or absolute abundance) drivers of ecosystem function, change in trait composition of communities has been identified as an undeniably important determinant of changes in ecosystem processes resulting from human disturbance [7]–[10]. From this research, the concept that human impacts on ecosystem functioning could be more effectively reversed with a mechanistic understanding of trait-based reassembly processes during habitat restoration arises. However, such a mechanistic approach to functional restoration remains completely unexplored.

Restoration ecology has long sought to identify the mechanisms that determine trajectories of community assembly [11]. More recently, trait-based ecology has provided a powerful approach to explore the mechanisms underpinning non-random assembly of communities [12], [13]. There has been some speculation on the likely functional consequences of varying community reassembly processes, such as the potential impact on provisioning of ecosystem services [14], [15]. However, there has been no experimental test of the utility of trait-based theory for determining the mechanistic link between community reassembly and the recovery of ecological processes.

One effective platform for linking species responses to environmental change with the functional consequences of shifting trait composition has been to employ a response-effect trait framework [16]. Although there has been a wealth of studies demonstrating the importance of functional traits in mediating both the response of species to environmental change and also their effects on ecosystem functioning [6], [9], [17], there have been no comparable response-effect trait studies that have investigated shifts in species trait composition within communities undergoing habitat restoration. Consequently, we are still unable to directly link the trajectory of community reassembly with the resulting recovery of ecosystem functioning. Nevertheless, response-effect trait models that combine significant recent advances in the biodiversity-ecosystem functioning debate [3] with assembly theory [18] and the development of trait-based theory in ecology [19] hold great promise for comprehensively understanding the processes that govern how ecosystem functioning will recover following habitat restoration.

In this study, we test the trait determinants of dung beetle community responses to experimental habitat restoration in the land-use matrix surrounding heavily degraded montane rainforest edges in Nigeria. Habitat loss, and subsequent degradation of rainforest edges due to cattle encroachment, fire, and altered biotic and abiotic conditions, are amongst the most severe drivers of biodiversity loss and alteration of ecosystem functioning in tropical forests [20], [21]. Edge effects, in particular, can have very strong effects on dung beetle communities [22], [23]. However, because of the trans-boundary nature of edge effects [24], there is the very real prospect of being able to mitigate edge effects by altering the type of matrix habitat that is adjacent to habitat remnants [22], [25]. Here, we explore the interactive effects of matrix restoration and edge effects on biotic communities [22] within a response-effect trait framework in order to gain insight into the complexity of factors that determine the recovery of ecosystem processes. We test the degree of importance of species functional traits for the recovery of ecosystem functioning, relating changes in trait distributions to variation in rates of dung removal, following the mitigation of anthropogenic threats through habitat restoration.

Although terrestrial invertebrates are the second most represented taxa in studies investigating trait-mediated ecosystem processes [10], there are few studies that take into account multiple effect traits and measures of trait divergence that might explain functional complementarity (e.g., [26], [27], [28]). Here, we identify the underlying mechanisms that mediate functional recovery of degraded ecosystems by testing the relative importance of three hypotheses for variation in dung removal rates: (1) species are functionally equivalent and ecosystem processes can be explained by purely biomass-dependent neutral process within a given trophic level; (2) over and above biomass-dependent effects, community-weighted trait means explain variation in relative rates of ecosystem function; and (3) ecosystem processing rates are explained by functional trait dispersion (community-level trait variability) via niche complementarity effects, independent of variation in community-weighted trait means. Using path modelling, we disentangle the interactions between multiple pathways of environmental impacts (edge effects and matrix habitat restoration) on dung removal, and demonstrate how trait structure and biomass mediate changes in ecosystem functioning in dung beetle communities undergoing restoration.

## Materials and Methods

### Study system

This study was conducted in Afromontane forest at the Ngel Nyaki Forest Reserve, located on the Mambilla Plateau near the south-eastern border of Nigeria (7.080234° N, 11.127765° E). No specific permissions were required regarding collection of invertebrate specimens in this location and our study did not involve any known endangered or protected species. The forest reserve is an outlying section of the West African montane forest network within the Cameroon Highlands ecoregion [29]. This region comprises a network of submontane forest remnants at elevations up to 2300 m, with a mean annual rainfall of approximately 1800 mm falling mostly during the April to October wet season, and mean monthly temperatures of 13 −26°C in the wet season and 16–23°C in the dry season [30]. Ngel Nyaki Forest Reserve is approximately 4600 ha and comprises a mosaic of overgrazed montane grasslands, degraded streamside forest/shrubland strips, and 720 ha of dense submontane forest [22].

As part of the Nigerian Montane Forest Project (NMFP) aimed at protecting Ngel Nyaki Forest Reserve from land clearing, burning and cattle grazing, three fenced exclusion zones were established up to 200 m outside the dense sub-montane forest zone, 3 years prior to the sampling procedure. These regenerating sites could then be compared with degraded edge zones where no restoration in the adjacent matrix had been established (S1 Figure). The length of fenced sections around the forest perimeter varied from 0.25 km to 1.6 km long. Within the 200 m fenced zone, cattle-grazing was eliminated and fire breaks were established within 2 m of each fence-line as a passive restoration strategy [15]. For two of the sampling transects within regenerating sites, the fenced area was too small to fit the entire edge gradient transect within the restoration zone in the matrix. As such, one transect extended only up to 40 m into the matrix and another extended up to 80 m, resulting in three missing sampling points. We used dung beetles as a focal taxon as they exhibit clear responses to environmental change and are directly responsible for the decomposition of dung detritus [31], [32], allowing direct measurements of ecosystem process rates carried out by these communities.

### Sampling protocol

Sampling was conducted at Ngel Nyaki Forest Reserve during the late rainy season from 4^th^ October to 29^th^ November 2009. To quantify the interactive effects of habitat edges and adjacent matrix restoration on dung beetle community structure and associated ecosystem processes, we sampled dung beetle communities and dung removal rates along three replicate forest-to-matrix edge gradients in both degraded and regenerating sites (n = 6). Although treatment-level replication was low, it is important to note that this is an experimental manipulation of matrix structure which was specifically targeted at a single experimental site where all edges had previously had a common history of edge degradation (just 3 years prior to sampling), and sampling completeness was high [22]. The common local context counters high site-to-site heterogeneity across the region that might otherwise bias interpretation. We acknowledge that with low treatment-level replication our study will only have the statistical power to detect ecological responses with large effect sizes [33], thus making our conclusions fairly conservative.

Degraded edges spanned forest-to-matrix habitats that were fully exposed to anthropogenic threats typical of the area (such as intensive cattle grazing and fires), compared to the regenerating edges where these threats had been entirely excluded for three years (S1 Figure). One additional ‘dummy edge gradient’ was placed in each of the forest interior and matrix interior habitats, at least 640 m from the forest edge, to test for potential spatial autocorrelation and capture rate interference among traps [34], from which we established that there was no support for such sampling effects [22]. Where possible, each replicate edge gradient consisted of up to 13 sampling points at fixed distances from the edge on a doubling scale (−160, −80, −40, −20, −10, −5, 0, 5, 10, 20, 40, 80, and 160 meters from the edge, where negative values represent forest samples). Traps were laterally offset from one another so that no two traps were closer than 50 m apart in order to maintain independence between traps (S2 Figure) as this distance has been suggested as a generic limit to which smaller-bodied dung beetles (such as the majority of those we sampled [S1 Table]) can detect dung [35]. This method of trap placement therefore reduced potential sampling bias from trap interference and spatial autocorrelation [34]. Furthermore, replicate edge gradients were at least 100 m apart, which was at least twice the distance that was maintained among sampling points within sites in order to avoid any interference among experimental variables (i.e. distance from edge and matrix restoration).

We used pitfall traps baited with 40 g of pig dung placed at each distance across the edge gradient for two consecutive 24 hour periods (pooled 48 hour samples for each edge gradient transect) to ensure adequate sampling of the local community [22]. Dung-baited pitfall traps consisted of 500 ml plastic cups with a depth of 11 cm and diameter of 8 cm, buried so that the rim of the cup was flush with the surface of the ground. To protect the trap from rain and falling debris, a wooden trap cover was held *ca* 20 cm above the cup using wooden stakes. From this trap cover, dung bait was suspended with string so that the bottom of the bait was level with the rim of the cup. The bait was contained within muslin mesh which allowed the scent of the bait to easily permeate into the surrounding atmosphere but was fine enough to exclude insects from directly accessing the bait and thus altering its attractiveness. The cup was filled with approximately 200 ml of water and five drops of highly concentrated, odourless, and clear detergent which served to break the surface tension of the water. Pig dung was used as bait because omnivore dung is recognised as the most widely attractive to dung beetles [36], was easily available, and also because wild pigs are common throughout Ngel Nyaki Forest Reserve. Domestic pigs were reared and fed a consistent controlled diet so that the dung used in the experiments was more likely to be chemically similar and thus similar in attractiveness regardless of the day it was collected. All traps for one entire edge sampling transect were set on a given day, and the order in which transects were sampled was randomised to avoid temporal autocorrelation. All dung beetles in the subfamily Scarabaeinae were sorted to genus and species [37] where possible, or assigned to ‘morphospecies’ groupings based on consistent morphological traits (see S2 Table).

### Quantification of dung removal rates

To quantify the impact of edge effects and matrix restoration on dung removal rates we placed experimental dung piles at each of the 101 sampling points and measured the proportion of dung removed in 24 hrs. Dung removal experiments were undertaken 1–2 days prior to baited pitfall trapping of dung beetles at each site, in order to avoid potential trap depletion effects on beetle communities that might otherwise have confounded dung removal rates. It should be noted that there could still have been potential interference of the removal experiment on pitfall trapping, as beetles that had already been attracted to dung placed out in the removal experiment may still have been buried in the soil, either laying eggs, provisioning brood balls, or already satiated. However, there is little reason to expect that this effect would have operated inconsistently across our treatments and is unlikely to have been systematically biased toward particular morphological traits of dung beetles, so we expect any such effect to have had a minor influence on our overall results. At each sampling point (as for pitfall trapping) debris such as dead wood or leaves within a 15 cm radius of dung placement was removed down to the topsoil and 40 g of fresh pig dung was placed directly on top of the bare soil. This amount of dung was sufficient to avoid complete removal and allow reliable comparisons among sampling points, but still equal to the amount of bait used in pitfall traps so that dung removal rates could be realistically compared to sampled dung beetle communities. After 24 hours, the remaining dung was collected and, after removing any attached debris, dry mass loss was calculated after taking into account moisture content loss, yielding a rate of dung removed per 24 hrs (see S1 Appendix).

### Measurement of functional traits and dung beetle biomass

To quantify variation in functional trait composition between communities at regenerating and degraded edge gradients, five morphological characteristics that are important response and/or effect traits in dung beetles [9], [38] were measured for individuals within each species: body mass, pronotum width, body shape index (BSI), wing area and wing loading. We restricted trait selection to these continuous morphological measurements so that quantitative causal relationships could be explored for all response and effect models without excluding potentially important intraspecific trait variation [39]. Body mass was calculated as the dry weight (mg) of each beetle and body size was estimated from the width (mm) of the pronotum. From these measures, we calculated BSI as the ratio of body mass to pronotum width. Wing area was calculated as the total area of the left hind wing (mm^2^), multiplied by two for total wing area, which was then used to calculate wing loading as the ratio of body mass to total wing area. To take into account within-species trait variation, we measured multiple individuals within each species for all samples collected. However, for highly-abundant species, we used a randomized subsampling procedure so that at least 20 individuals were measured per sample for each abundant species (see S2 Appendix, S3 Figure, S1 Table). In order to estimate the total biomass of dung beetles for each sample, we summed individual body mass measurements from all specimens in a given sample.

### Statistical Analysis

#### Using continuous response functions to quantify functional effects of matrix restoration

Variation in the total biomass of dung beetles and rates of dung removal were analyzed across forest-to-matrix gradients for both degraded and regenerating matrix treatments using the statistical approach of Ewers & Didham [40]. Using a form of the general logistic model we determined the best-fit edge model out of five models of increasing complexity (see S3 Appendix).

#### A multilevel path model to disentangle causal pathways of functional restoration

**Figure 1:**
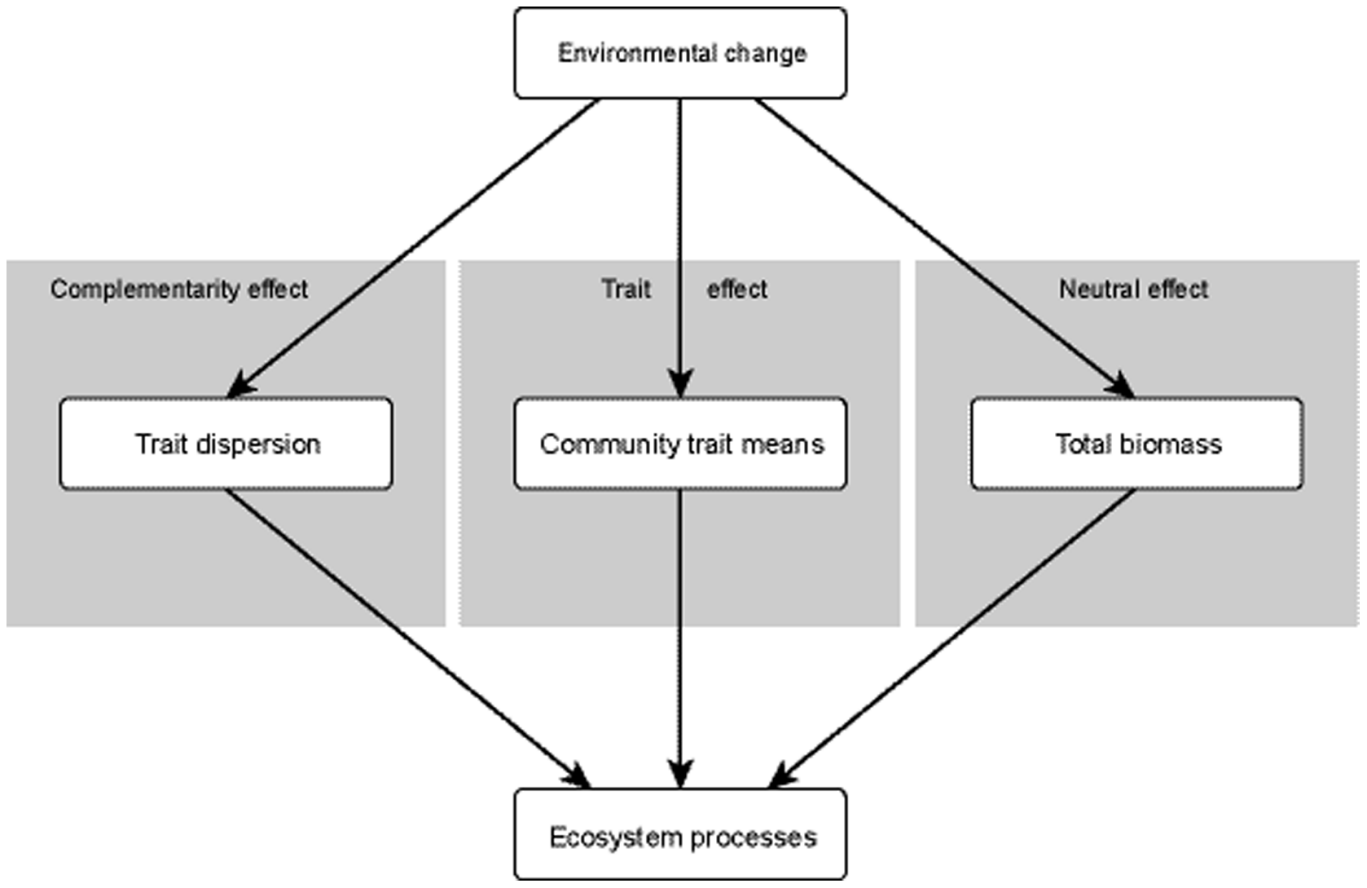
Hypothetical causal pathways of the effects of environmental change on insect-mediated ecosystem processes. Shaded boxes indicate the causal hypotheses (complementarity effect, trait-mean effect, or neutral effect).

To determine whether any correlation between beetle community structure and dung removal was driven by purely neutral, biomass-dependent processes (i.e. total beetle biomass irrespective of species identity) or by variation in the abundances of species with differing traits, we used a hierarchical path modelling approach [41] in R 3.0.2 [42] (see S4 Appendix). To test trait versus neutral effects in dung beetle-mediated dung decomposition, we partitioned potential explanatory pathways into three main hypotheses (Fig. 1). First, rates of dung removal might be entirely dependent on total dung beetle biomass. We use total biomass here because metabolic zero-sum dynamics, which are central to Hubbell's [43] model of neutral theory, can be explicitly characterised by the regulation of consumer biomass by absolute energy availability in a system [44]. As such, total biomass provides a measure of per mg resource assimilation by dung beetle communities under the assumptions of metabolic zero-sum dynamics, but irrespective of individual effect traits.

Second, dung removal rates might be dependent on average trait values expressed in a given community. To test this hypothesis, we included all measured traits (i.e. body mass, pronotum width, wing area, wing loading, and BSI) within the path model. Because we suspected there could be collinearity among the trait predictors, we checked for correlations among variables while constructing the path model. In most cases, predictors within the GLMMs were sufficiently weakly correlated so that interpretation of the models was considered reliable (|r|<0.7) [45]. In the few cases where predictor correlations exceeded this threshold, we re-ran models following the sequential exclusion of correlated trait predictors in order to validate model performance. From this procedure, we established that there was no qualitative change in the overall structure of the path model due to collinearity, so we retained the full comparison of multiple traits in the path model. We believe that this provides more reliable and comprehensive interpretation than if correlated traits are arbitrarily excluded.

Third, we hypothesised that there might be a niche complementarity effect whereby community functional trait dispersion determines dung removal efficiency of dung beetle communities. As a measure of functional trait complementarity, we calculated a distance-based metric of trait functional dispersion (FDis) using the “FD” package [46] in R 3.0.2 [42]. The FDis metric takes into account multiple trait characteristics of organisms within a community and measures the distance of each species to the trait-mean centroid of the whole community. It is a multivariate adaptation of weighted mean absolute deviation from the trait centroid, where the weighting is given by the relative abundance of species [46]. It is thus a weighted measure of trait variation or complementarity among species in a given community. To calculate FDis, we first compiled a trait matrix with mean trait values for each species, then calculated Gower dissimilarity coefficients among species trait complexes using the “gowdis” function. This was used to determine multivariate dispersion of assemblages based on the Gower dissimilarity coefficients weighted by species relative abundances.

## Results

### Matrix regeneration alters beetle communities and associated ecosystem functioning

**Figure 2:**
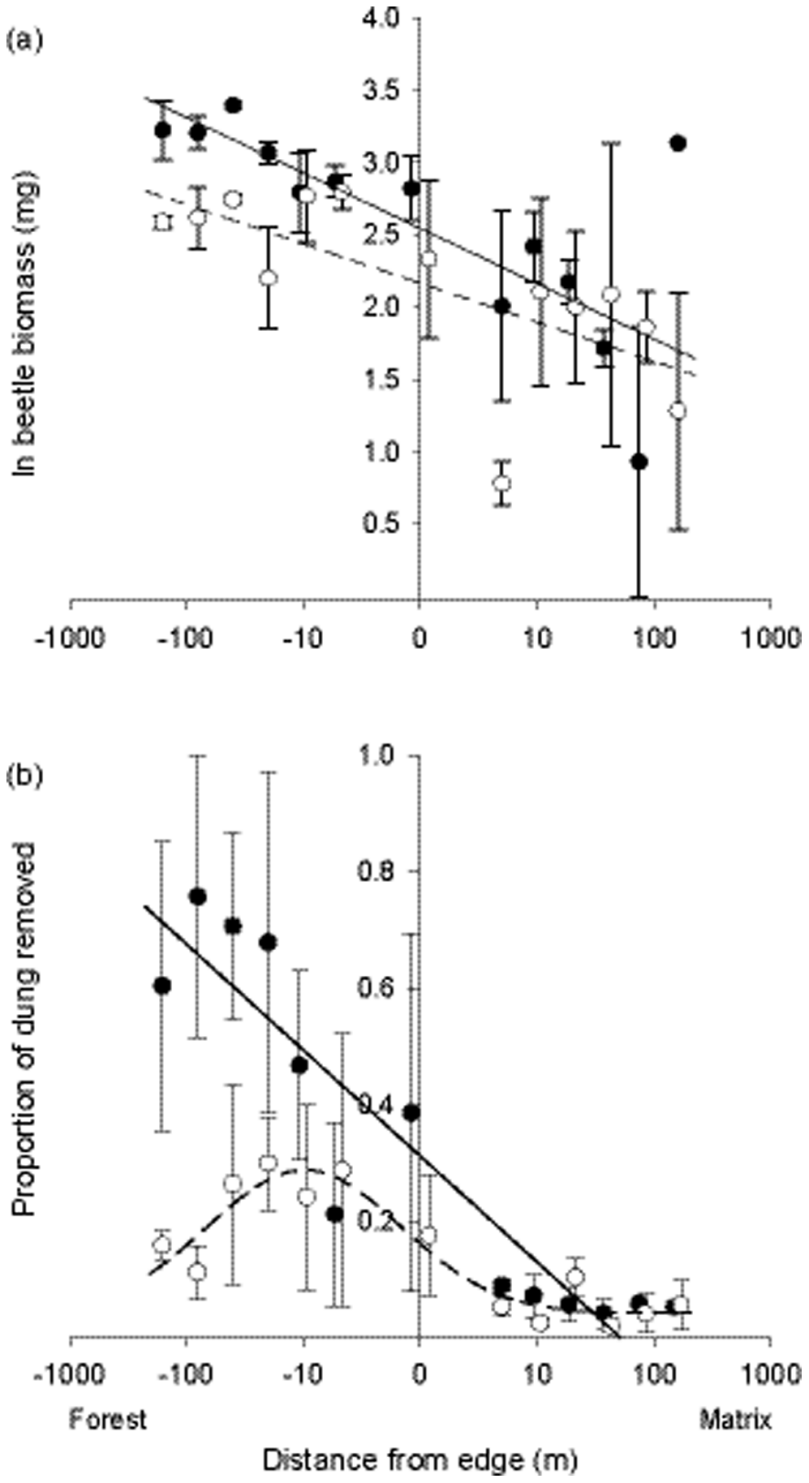
Variation in total beetle biomass and proportion of dung removed across degraded and regenerating edges. Points and error bars are mean ±1 SE. Open symbols and dashed fitted lines denote degraded edges, whereas closed symbols and solid fitted lines denote regenerating edge gradients. Negative values on the x-axis indicate forest sites. Lines are the best-fit continuous edge response functions of five fitted models of increasing complexity. Both model fits in (a) are linear, while the model fit for regenerating edges in (b) is linear and for degraded edges in (b) is unimodal (see Appendix S3). Overlapping points are offset for clarity.

A total of 4705 dung beetles were captured across all sites, comprising 33 species in 12 genera (S2 Table). Of these, 28% of species were captured exclusively in forest habitat and 42% were restricted to matrix habitats. There was a negative relationship between the distance from edge and total biomass of dung beetles across forest-to-matrix gradients (Fig. 2a, S3 Table). However, there appeared to be only a weak influence of matrix restoration on dung beetle biomass responses to habitat edge effects, except in the forest interior (Fig. 2a, S3 Table).

Dung removal rates also varied dramatically across habitat edge gradients, ranging from an average of >75% dung removal over a 24-hr period in the forest interior to ∼0% removal in the matrix habitat (Fig. 2b, S3 Table). Moreover, despite the relatively modest effect of matrix regeneration on total beetle biomass, there was a very strong effect of adjacent matrix regeneration on dung removal rates (Fig. 2b). At forest sites adjacent to regenerating matrix there was up to a 6 fold increase in dung removal compared to degraded forest sites (Fig. 2b). Of particular interest was the apparent off-site effects of adjacent matrix restoration, as there were only marked increases in dung removal rates within the forest and not in the regenerating matrix itself (Fig. 2b).

### Determining pathways of beetle-mediated ecosystem processes

**Figure 3:**
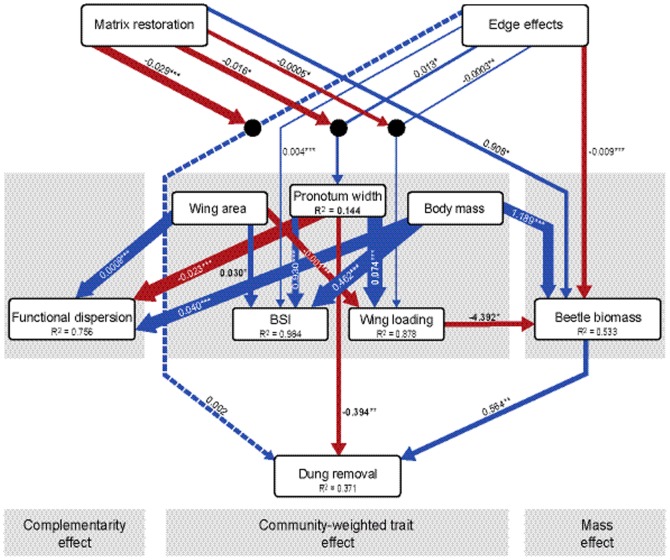
Causal pathways of functional responses to edge effects and matrix restoration. Best-fit generalized multilevel path model structure (χ^2^ = 44.964, df = 54, P = 0.805) as determined by the ***B*** *_U_* set of independence claims (Table S4). Boxes depict predictor and response variables and arrows indicate significant positive (blue) and negative (red) effects, with non-significant effects indicated by a dashed arrow. Circular nodes with arrows leading to them represent interaction terms. R^2^ values in the endogenous variables indicate the strength of fit for individual models. Values within and adjacent to arrows are the unstandardized path coefficients with level of significance (* p<0.05, ** p<0.01, *** p<0.001). Line weightings indicate the relative strength of effects as derived from the standardized path coefficients.

Results from the multilevel path analysis revealed that dung removal rates were influenced by trait-dependent effects, over and above the positive influence of total beetle biomass on removal rates (Fig. 3). First, the total biomass of dung beetles decreased significantly from forest to matrix habitats and was significantly higher in regenerating habitats. As expected, total dung beetle biomass was also positively influenced by mean body mass of constituent dung beetle species within samples. However, total biomass decreased significantly with increasing community-weighted mean wing loading in dung beetle communities (Fig. 3). Together, these factors explained 53% of the variation in beetle biomass, and these biomass-dependent neutral effects had a positive influence on dung removal rates (standardized effect size 0.342±0.112, P<0.01).

Second, community-weighted trait composition also responded significantly to both edge effects and matrix restoration, but with variable responses across different traits. For instance, at degraded edge gradients there was a significant increase in mean pronotum width, wing loading and BSI of individual species from the forest into the matrix habitats (Fig. 3). However, for pronotum width and wing loading there was a significant interaction effect between edge influence and matrix restoration, which reduced the intensity of edge responses in community-weighted trait distributions at the regenerating edge gradients (Fig. 3). Surprisingly, however, there was no significant impact of edge effects or matrix restoration on either body mass or wing area. Of all the observed response traits, most had no discernible functional consequences for dung removal rates. Only the combined influences of edge effects and matrix restoration on community-weighted pronotum width translated into a significant negative influence on dung removal (standardized effect size −0.319±0.109, P<0.01). After controlling for the positive effect of total dung beetle biomass on dung removal rates, we found that sites which had smaller beetle species, on average, tended to have higher dung removal rates (Fig. 3 and S4 Figure).

As expected, variation in the trait-mean composition of species strongly influenced functional trait dispersion, particularly with respect to wing area, body mass, and pronotum width effects, together explaining 76% of variation in functional dispersion across samples. For our study system, however, there were no apparent direct influences of edge effects or matrix restoration on functional trait dispersion after controlling for variation in community-weighted trait composition. There was also no flow-on effect of functional trait dispersion on rates of dung removal, despite the strong association of trait composition with dispersion.

Finally, the relative partitioning of trait mean effects, functional trait dispersion, and mass-dependent neutral effects did not capture all the potential proximate factors mediating the effect of anthropogenic disturbance on dung removal rates. There was a significant interaction effect between matrix restoration and edge effects that had a residual direct influence on dung removal rates at matrix restoration sites, although at degraded sites there was no residual direct effect of edge impacts on dung removal rates (over and above the effects of community-weighted pronotum width and total biomass) (Fig. 3). This suggests that unmeasured mechanisms, beyond variation in dung beetle community attributes, led to a significantly greater forest-to-matrix difference in dung removal rates following matrix restoration, than observed at degraded edges (Fig. 3).

## Discussion

We demonstrate that matrix habitat restoration can have a profound influence on nutrient cycling-related ecosystem functioning at degraded tropical forest edges. For the removal of dung by dung beetles, the path to functional recovery depended not only on the random reassembly processes contributing to total dung beetle biomass (irrespective of functional trait identities), but also on the body sizes (i.e. pronotum widths) of recolonizing individuals. Given the importance of restoring ecosystem functioning in restoration efforts [15], our study provides valuable insight into the mechanisms underpinning functional recovery, and suggests that relatively simple restoration efforts in matrix habitat can be highly effective in mitigating anthropogenic impacts on community trait composition, biomass, and associated ecosystem processes in adjacent forest remnants.

The key to determining the pathways through which matrix restoration drives functional recovery was the application of a response-effect trait framework within a path-modelling context. While this approach has been widely adopted in modelling human impacts on ecosystem processes within degraded systems, it holds untapped promise in a restoration context. From this analysis, we showed that matrix restoration substantially ameliorated the negative impacts of habitat edge effects on dung beetle biomass and community trait composition observed between forest and matrix habitats at the degraded sites. In particular, edge effects on community-weighted trait means of dung beetle pronotum width and wing loading at degraded edges were significantly reduced by the restoration of the adjacent matrix habitat. Given that small-bodied invertebrate species typically have lower physiological tolerance to anthropogenic disturbance [47], and species with low dispersal ability should be more restricted in crossing hostile environments [38], [48], these results tend to suggest that matrix restoration was highly successful in facilitating the recovery of disturbance-sensitive species with smaller average body size and lower wing loading. Moreover, these species contributed substantially to the higher total biomass of dung beetle communities observed in the regenerating matrix sites.

In many ways, this rapid rate of recovery is surprising after just three years of experimental matrix regeneration. Many previous studies have suggested there can be long lag-times to faunal community re-assembly following revegetation, particularly for small-bodied species with low dispersal capacity [49], [50], but see [51], [52]. In this study, we were not able to determine the exact mechanisms that drove this increase in total biomass and decreasing average body size of dung beetle communities at regenerating edges. However, it is likely that the restored matrix zones adjacent to the Ngel Nyaki Forest Reserve act as habitat buffers against anthropogenic disturbances from the degraded matrix. Therefore, species that are sensitive to edge effects across degraded edges [22] might be preferentially moving to (or increasing reproductive output in) forest areas that are buffered by zones of habitat restoration in the adjacent matrix.

Surprisingly, matrix habitat restoration had no direct influence on community-wide trait dispersion at forest edges, but there were significant indirect effects observed via the mediating effects of community-weighted trait means on functional trait dispersion. In particular, there were highly significant effects of community-weighted mean body mass, pronotum width, and wing area on functional trait dispersion, with all three traits having equivalent standardized effect sizes. Because the vast majority of dung beetles captured (>86%) had a body mass of <10 mg (despite maximum body mass of 1543.07 mg) and wing area of <20 mm^2^ (despite maximum wing area of 1098.53 mm^2^), the key driver of variation in trait dispersion was the distribution of the few rare beetles with large body mass and relatively large wing area (after accounting for variation in body mass in the partial regression relationships). Interestingly, there was a significant negative effect of community-weighted pronotum width on functional dispersion (after accounting for variation in body mass in the partial regression relationships). We interpret this as species that are smaller than expected based on their body mass making a greater contribution to high trait dispersion.

By partitioning community-wide responses into separate trait-mean variables versus overall variability in community-level trait dispersion, our results demonstrate the varying sensitivity of different trait measures to environmental change. Functional trait dispersion was strongly affected by matrix habitat restoration at forest edges, but these effects were only manifested indirectly via the shared influence on multiple components of trait variation. No single trait response variable could explain the observed response in functional trait dispersion in its own right, suggesting the need to quantify multiple traits in order to capture their role in community assembly during restoration. At the same time, though, only very few trait responses were required (three in this case) to explain a relatively high proportion of the variation (76%) in community-wide trait dispersion.

Trait determinants of community responses to environmental change also had a significant influence on rates of beetle-mediated dung removal. Although we found no niche complementarity effect on dung removal driven by variation in functional trait dispersion, there was a clear effect of community-weighted mean trait composition on dung removal rates, over and above neutral mass-dependent effects. This was demonstrated by the relatively large standardised effect size of community-weighted mean pronotum width on dung removal (−0.319), which had almost as strong a standardised effect on dung removal as total beetle biomass (0.342), supporting the claim that neutral processes alone may not be able to fully explain functional processes [53]. While the path model employed in this study provides insight into the relative importance of different morphological traits for functional efficiency of dung beetles, it is important to bear in mind that functional responses stem from variation in suites of collinear (and often coevolved) traits. Single-trait explanations for responses should be treated with some caution due to the collinearity of the traits measured. Nevertheless, our approach does clearly demonstrate that the trait characteristics of species (such as body size) are important determinants of both responses and functional effects of dung beetles across regenerating forest edges.

Surprisingly, the mediating effect of community-weighted mean pronotum width on dung removal was negative, suggesting that in samples with a smaller weighted-average body size of dung beetles, the removal rate of dung was proportionately greater per unit mass of beetles. Many previous studies have pointed to the importance of large dung beetles in dung decomposition rates, whereby body size is assumed to be positively correlated with amount of dung sequestered [28], [35], [54]. However, previous studies have not quantified ‘gram for gram’ beetle-to-dung weight ratios of removal efficiency. As a result, our findings indicate that if total community biomass is held constant, communities composed of smaller dung beetles, on-average, are more likely to perform higher rates of dung removal. While the mechanisms that determine this result have not been explicitly tested in our study, we suggest that this negative relationship between individual body mass and removal efficiency (while holding total biomass constant) can be clearly explained by the metabolic theory of ecology [55]. Specifically, because the relationship between body size and individual whole-organism metabolic rate is non-linear (i.e. scales according to the 3/4-power scaling law), this means that smaller-bodied organisms have higher mass-specific metabolic rates compared to larger organisms [55]. Therefore, smaller organisms tend to have higher metabolic demand per unit mass. As such, if total biomass is held constant, the total metabolic demand of communities composed of smaller organisms should be higher than communities composed of larger organisms. This could explain why we found the negative relationship between body size and dung removal rates, when holding total biomass constant. Interestingly, Nichols *et al*. [56] recently found a similar positive relationship between high biomasses of small-bodied dung beetles and burial of seeds within dung, supporting our findings that small-bodied beetles may in fact contribute more than previously expected to overall ecosystem functioning. Bearing that in mind, community-weighted mean body mass was also associated with an increase in overall total beetle biomass and therefore still conferred an indirect positive effect on removal rates. Taken together, it is apparent that the effect of matrix habitat restoration on dung removal occurs through multiple mechanisms, with restoration leading to greater total biomass composed of smaller beetles that appear to perform higher per-unit-mass removal of dung, together resulting in higher overall dung removal rates.

In addition to mass- and trait-dependent effects, we also detected a significant residual interaction effect of our treatment drivers on overall rates of dung removal, with matrix restoration mitigating the low rates of dung removal observed at degraded edges significantly more than could be explained by recovery in dung beetle biomass or trait-dependence in reassembly processes alone. This is almost certainly due to unmeasured variation in environmental parameters along edge gradients, such as substantial reduction in dung desiccation rates at regenerating edges (which could alter dung attractiveness) and facilitation or competition from other dung-associated organisms that are likely to alter removal rates differentially among regenerating and degraded edge gradients. Furthermore, it is possible that other unmeasured traits such as dung removal strategy or diel activity patterns could help to explain some of this residual variation. A better mechanistic understanding of these processes is still needed in order to understand how other contributing factors such as these might help to explain variation in ecosystem functioning following restoration.

### Conclusions

Overall, this experiment has shown that restoration of the matrix surrounding degraded tropical forest remnants can drive large increases in the biomass of organisms and their associated ecosystem processes, even over very short time periods. Interestingly, the enhancement of dung removal rates through restoration could not be explained solely as a function of increasing biomass of decomposer organisms without recourse to trait-dependence in ecosystem process rates. A notable proportion of variation in dung removal was explained by community-mean body size that in turn resulted in significant effects on dung removal, suggesting that ‘neutral’ measures of community assembly alone cannot explain functional outcomes of habitat restoration. Rather, we found that recovery of a suite of disturbance-sensitive species with low dispersal power and small body size, but high per capita dung removal efficiency (for their size), resulted in higher dung removal rates at habitat edges undergoing adjacent matrix restoration. The observed mediating effects of response and effect traits on dung removal are likely to have far-reaching consequences for heavily-degraded tropical forest remnants, through cascading changes in insect-mediated ecosystem functions such as nutrient cycling rates and secondary seed dispersal that can have strong deterministic impacts on plant communities [57], [58]. As such, the rapid recovery in biomass and trait-mean composition observed after just three years of fencing, fire-exclusion and revegetation brings with it the very real prospect that matrix habitat restoration can mitigate land-use impacts and restore biodiversity and ecosystem functioning to tropical forest remnants.

## Supporting Information

S1 Figure **Layout of edge gradient sampling transects.**
(DOCX)

S2 Figure **Edge-gradient sampling design.**
(DOCX)

S3 Figure **Example of the left hind wing of an individual male Onthophagus sp. 1.**
(DOCX)

S4 Figure **Contour plot demonstrating the combined effects of total beetle biomass and community-weighted mean body mass on proportion of dung removed.**
(DOCX)

S1 Table **Trait mean values for dung beetle species.**
(DOCX)

S2 Table **List of dung beetle species and their occurrences.**
(DOCX)

S3 Table **Akaike Information Criterion (AIC) scores obtained from the edge function fitting procedure.**
(DOCX)

S4 Table **Complete basis set of independence claims for the selected best-fit path model.**
(DOCX)

S1 Appendix **Measuring rates of dung removal.**
(DOCX)

S2 Appendix **Measurement of dung beetle traits.**
(DOCX)

S3 Appendix **Continuous edge response models.**
(DOCX)

S4 Appendix **Constructing generalised multilevel path models.**
(DOCX)
