# Supplementary material for: The Role of Species Traits in Mediating Functional Recovery during Matrix Restoration
Source: PLoS One. 2014 Dec 12;9(12):e115385. doi: 10.1371/journal.pone.0115385 (PMC4264948; doi:10.1371/journal.pone.0115385)

**Figure S1. Layout of edge gradient sampling transects.** Satellite image of the western perimeter of Ngel Nyaki Forest Reserve with forest depicted by dark gray and pastoral matrix by light grey. The locations of edge gradient sampling transects are marked with solid and dashed lines for regenerating and degraded matrix sites, respectively (actual spatial layout of edge gradient sampling sites are explained in Appendix S2). Fences and fire breaks are marked by the dark grey lines in the matrix adjacent to the regenerating edge gradient transects. Image was aquired from Ngel Nyaki Forest Reserve with the Quickbird satellite on January 9, 2009.


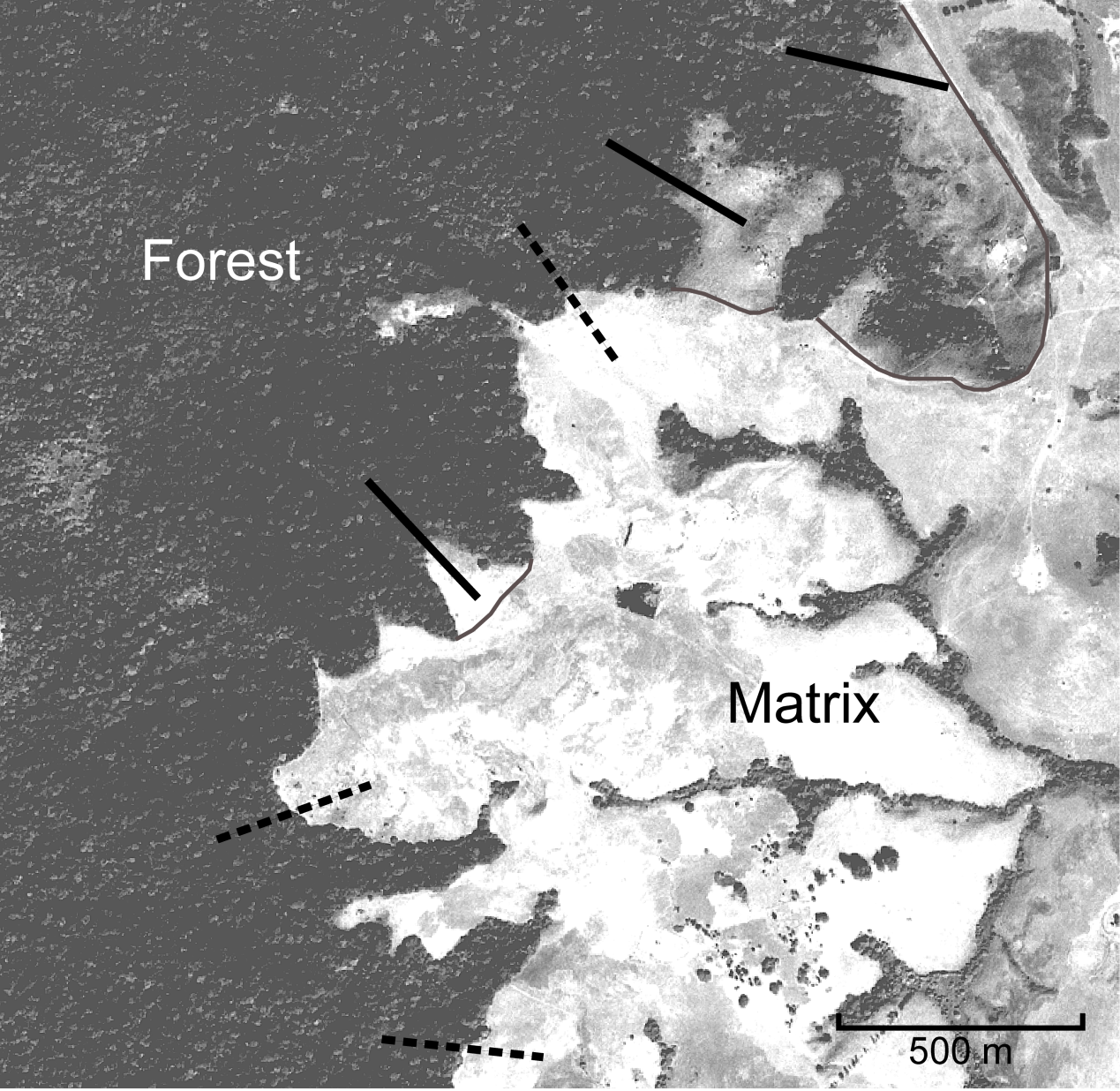

Supplement: S1 Figure — Layout of edge gradient sampling transects. (DOCX) [file pone.0115385.s001.docx]
