# Supplementary material for: The Role of Species Traits in Mediating Functional Recovery during Matrix Restoration
Source: PLoS One. 2014 Dec 12;9(12):e115385. doi: 10.1371/journal.pone.0115385 (PMC4264948; doi:10.1371/journal.pone.0115385)
Supplement: S2 Figure — Edge-gradient sampling design. (DOCX) [file pone.0115385.s002.docx]

Figure S2. Edge-gradient sampling design. Illustrative depiction of the spatial layout of one of the six edge-gradient sampling sites. Circles denote dung-baited pitfall traps in both the forest and matrix habitats with lateral displacement to maintain a minimum distance of 50 m between each trap. The dashed line denotes the forest edge, and the doubling scale on the right indicates the relative distance of each trap in meters from the forest edge.

*
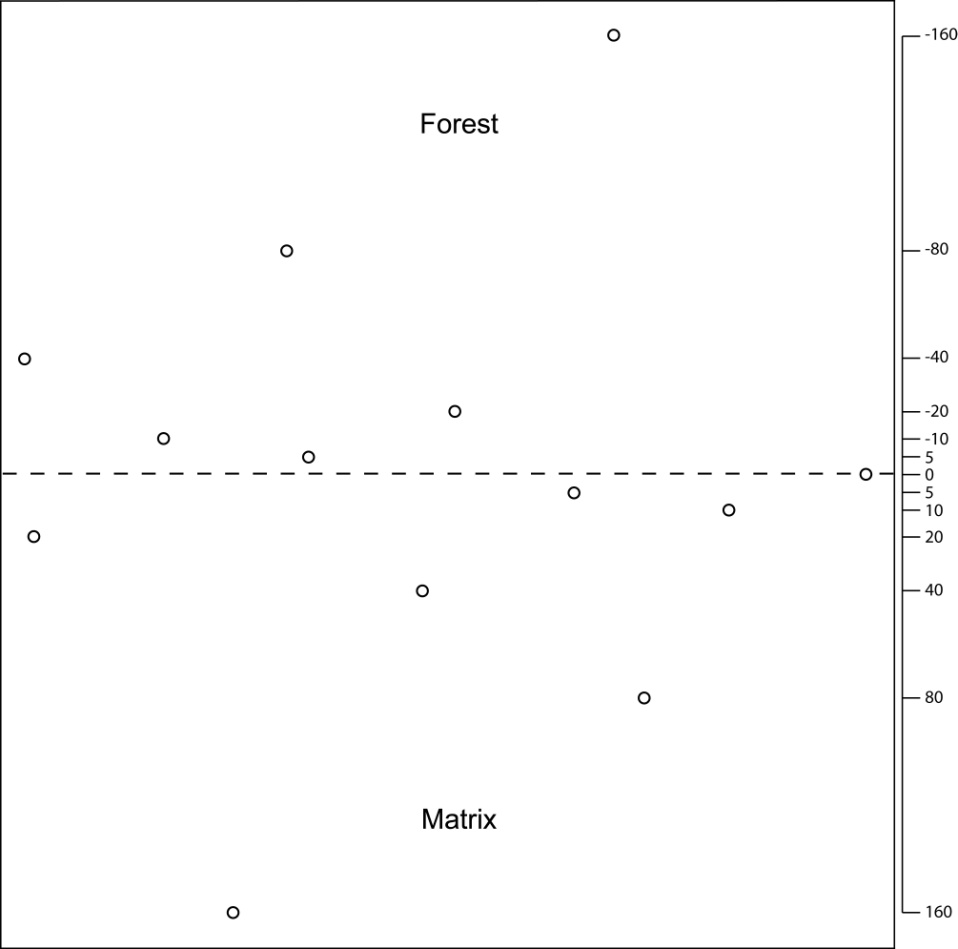
*
