# Supplementary material for: The Role of Species Traits in Mediating Functional Recovery during Matrix Restoration
Source: PLoS One. 2014 Dec 12;9(12):e115385. doi: 10.1371/journal.pone.0115385 (PMC4264948; doi:10.1371/journal.pone.0115385)

Figure S3. Example of the left hind wing of an individual male *Onthophagus* sp. 1.

Red arrows mark the two vein juncture landmarks used to orient a planar cut-off point (black dashed line) for standardisation of area measurements.


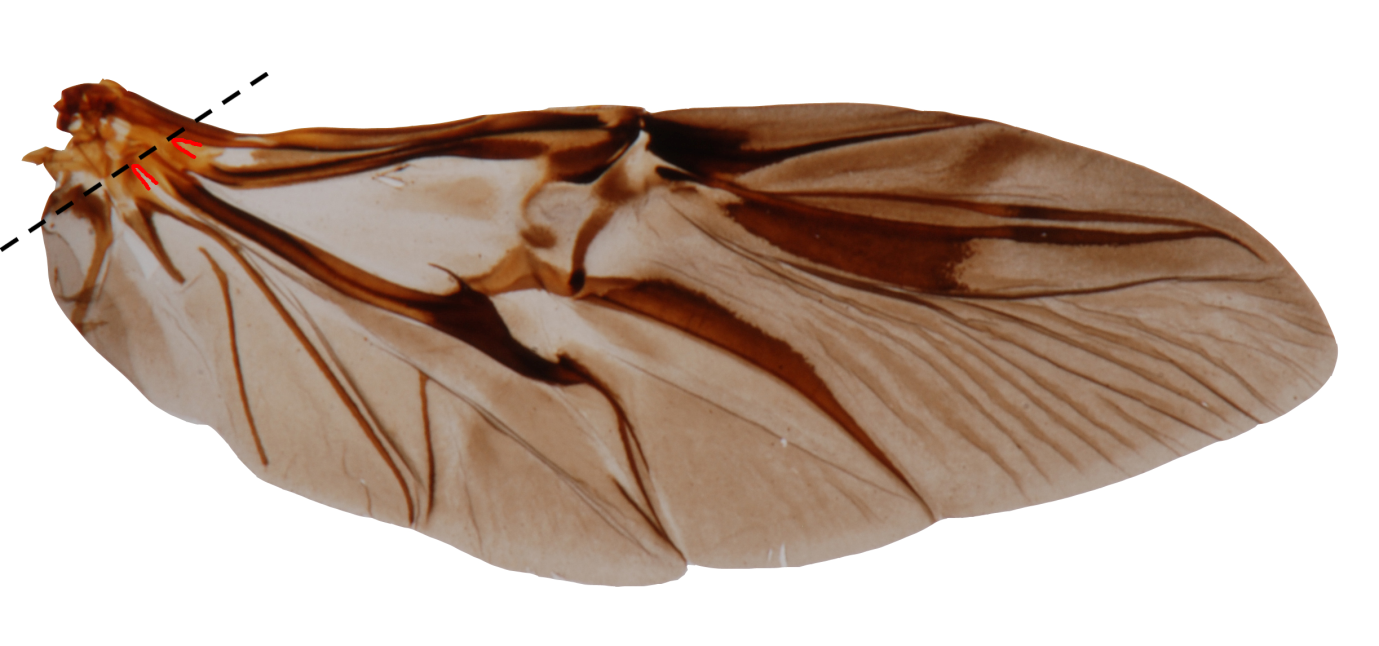

Supplement: S3 Figure — Example of the left hind wing of an individual male Onthophagus sp. 1. (DOCX) [file pone.0115385.s003.docx]
