# Supplementary material for: The Role of Species Traits in Mediating Functional Recovery during Matrix Restoration
Source: PLoS One. 2014 Dec 12;9(12):e115385. doi: 10.1371/journal.pone.0115385 (PMC4264948; doi:10.1371/journal.pone.0115385)
Supplement: S4 Figure — Contour plot demonstrating the combined effects of total beetle biomass and community-weighted mean body mass on proportion of dung removed. (DOCX) [file pone.0115385.s004.docx]

**Figure S4. Contour plot demonstrating the combined effects of total beetle biomass and community-weighted mean body mass on proportion of dung removed.** Proportion of dung removed is indicated by the colour fill. At very low levels of total beetle biomass, it seems self-evident that larger beetles will remove larger quantities of dung than smaller beetles. Surprisingly, however, at sites with high total beetle biomass, the highest observed dung removal rates were for communities with smaller average body mass.
