# Supplementary material for: The Role of Species Traits in Mediating Functional Recovery during Matrix Restoration
Source: PLoS One. 2014 Dec 12;9(12):e115385. doi: 10.1371/journal.pone.0115385 (PMC4264948; doi:10.1371/journal.pone.0115385)
Supplement: S1 Table — Trait mean values for dung beetle species. (DOCX) [file pone.0115385.s005.docx]

Table S1. Trait mean values for dung beetle species. Values are the arithmetic means calculated from all measured individuals (out of the total number of specimens captured, in parentheses). Body shape index (BSI) = body mass / pronotum width. Wing loading = body mass / wing area. Four individuals were excluded from the total numbers as they were damaged and traits could not be measured.

| **Species** | **Body mass**  **(mg)** | **Pronotum width (mm)** | **Wing loading** | **BSI** | **Individuals measured** |
| --- | --- | --- | --- | --- | --- |
| *Caccobius pentagonus* d'Orbigny | 1.197 | 2.053 | 0.166 | 0.579 | 4 (4) |
| *Caccobius ocellipennis* d’Órbigny | 1.159 | 2.031 | 0.244 | 0.571 | 1 (1) |
| *Catharsius dux* Harold | 930.099 | 19.980 | 1.416 | 46.109 | 11 (12) |
| *Catharsius* sp.n. | 78.211 | 9.566 | 0.514 | 8.175 | 7 (7) |
| *Catharsius sesostris* Waterhouse | 136.363 | 12.274 | 0.582 | 11.110 | 1 (1) |
| *Diastellopalpus nigerrimus* Kolbe | 224.999 | 12.037 | 1.968 | 18.516 | 65 (65) |
| *Heliocopris myrmidon* Kolbe | 1543.067 | 25.725 | 1.700 | 59.868 | 3 (3) |
| *Latidrepanus caelatus* Gerstaecker | 2.786 | 2.528 | 0.384 | 1.098 | 40 (40) |
| *Liatongus arrowi* Boucomont | 15.674 | 4.503 | 0.752 | 3.481 | 1 (1) |
| *Neosisyphus armatus* Gory | 19.178 | 4.636 | 0.918 | 4.095 | 6 (6) |
| *Onitis fabricii* Roth | 214.119 | 12.786 | 1.688 | 16.680 | 10 (10) |
| *Onitis* sp.n. aff. fabricii | 133.414 | 10.596 | 1.676 | 12.591 | 1 (1) |
| *Onthophagus* sp.n. 1 | 29.861 | 6.022 | 0.684 | 4.920 | 198 (198) |
| *Onthophagus* sp.n. 2 | 7.777 | 3.619 | 0.474 | 2.131 | 1294 (2130) |
| *Onthophagus* sp.n. 3 | 2.910 | 2.757 | 0.322 | 1.058 | 1006 (1787) |
| *Onthophagus* sp.n. 4 | 3.018 | 2.715 | 0.214 | 1.111 | 4 (4) |
| *Onthophagus* sp.n. 5 | 2.597 | 2.529 | 0.384 | 1.005 | 27 (27) |
| *Onthophagus* sp.n. 6 | 2.172 | 2.468 | 0.290 | 0.871 | 121 (163) |
| *Onthophagus* sp.n. 7 | 5.187 | 3.048 | 0.322 | 1.666 | 24 (24) |
| *Onthophagus fuscidorsis* d'Orbigny | 10.681 | 3.885 | 0.586 | 2.706 | 35 (35) |
| *Onthophagus juvencus* Klug | 7.928 | 3.656 | 0.444 | 2.167 | 7 (7) |
| *Onthophagus marginifer* Frey | 3.866 | 3.204 | 0.380 | 1.206 | 41 (41) |
| *Onthophagus* indet. sp. 11 | 3.565 | 2.958 | 0.266 | 1.133 | 6 (6) |
| *Onthophagus rufonotatus* d’Orbigny | 21.990 | 4.989 | 1.104 | 4.385 | 14 (14) |
| *Onthophagus* sp.n. 8 | 3.850 | 2.905 | 0.392 | 1.318 | 31 (31) |
| *Onthophagus jacksoni* d’Orbigny | 3.087 | 2.723 | 0.404 | 1.132 | 7 (7) |
| *Onthophagus* sp.n. 9 | 11.207 | 4.478 | 0.338 | 2.508 | 7 (7) |
| *Onthophagus longipilis* d’Orbigny | 11.220 | 3.777 | 0.780 | 2.949 | 9 (9) |
| *Onthophagus bidentifrons* d’Orbigny | 1.453 | 2.693 | 0.174 | 0.541 | 2 (2) |
| *Onthophagus* sp. 18 | 2.089 | 2.561 | 0.294 | 0.816 | 1 (1) |
| *Proagoderus elgoni* d'Orbigny | 53.673 | 7.702 | 1.254 | 6.829 | 14 (14) |
| *Proagoderus multicornis* d'Orbigny | 60.012 | 7.895 | 1.128 | 7.512 | 9 (9) |
| *Sisyphus* indet. sp. 1 | 2.740 | 2.720 | 0.334 | 1.000 | 29 (29) |
