# Supplementary material for: The Role of Species Traits in Mediating Functional Recovery during Matrix Restoration
Source: PLoS One. 2014 Dec 12;9(12):e115385. doi: 10.1371/journal.pone.0115385 (PMC4264948; doi:10.1371/journal.pone.0115385)
Supplement: S2 Table — List of dung beetle species and their occurrences. (DOCX) [file pone.0115385.s006.docx]

**Table S2.** **List of dung beetle species and their occurrences**. Occurrences are shown across habitat (forest and matrix) and adjacent matrix type with the total number of individuals per species. Note that for the genus *Sisyphus*, there is potentially an unresolved complex of two species that have been analysed as a single morphospecies. Reference collections are held at the Denver Museum of Nature and Science, USA, and the Department of Entomology, Lincoln University, New Zealand.

|  |  | Regenerating matrix sites | |  | Degraded matrix sites | |  |  |
| --- | --- | --- | --- | --- | --- | --- | --- | --- |
| Species | Forest control | Forest | Matrix |  | Forest | Matrix | Matrix control | Total |
| *Caccobius pentagonus* d'Orbigny |  |  | 3 |  |  | 1 |  | 4 |
| *Caccobius ocellipennis* d’Órbigny |  |  | 1 |  |  |  |  | 1 |
| *Catharsius dux* Howard |  |  | 2 |  |  | 3 | 7 | 12 |
| *Catharsius* sp.n. | 4 | 1 |  |  | 2 |  |  | 7 |
| *Catharsius sesostris* Waterhouse |  |  |  |  |  | 1 |  | 1 |
| *Diastellopalpus nigerrimus* Kolbe | 1 | 50 | 1 |  | 13 |  |  | 65 |
| *Heliocopris myrmidon* Kolbe |  |  | 2 |  |  | 1 |  | 3 |
| *Latodrepanus caelatus* Gerstaecker |  | 9 | 27 |  | 1 | 3 |  | 40 |
| *Liatongus arrowi* Boucomont |  | 1 |  |  |  |  |  | 1 |
| *Neosisyphus armatus* (Gory) |  |  | 2 |  |  | 2 | 2 | 6 |
| *Onitis fabricii* Roth |  |  |  |  |  | 10 |  | 10 |
| *Onitis* sp.n. (aff. fabricii) |  |  |  |  |  | 1 |  | 1 |
| *Onthophagus* sp. n. 1 | 17 | 133 | 1 |  | 47 |  |  | 198 |
| *Onthophagus* sp. n. 2 | 118 | 1338 | 20 |  | 655 |  |  | 2131 |
| *Onthophagus* sp. n. 3 |  | 1044 | 115 |  | 624 | 4 |  | 1787 |
| *Onthophagus* sp. n. 4 |  | 1 |  |  | 3 |  |  | 4 |
| *Onthophagus* sp. n. 5 |  | 1 | 15 |  |  | 6 | 5 | 27 |
| *Onthophagus* sp. n. 6 |  | 4 | 148 |  | 5 | 4 | 3 | 164 |
| *Onthophagus* sp. n. 7 | 1 | 17 |  |  | 5 | 1 |  | 24 |
| *Onthophagus fuscidorsis* d'Orbigny | 17 | 8 |  |  | 10 |  |  | 35 |
| *Onthophagus juvencus* Klug |  |  | 1 |  |  | 4 | 2 | 7 |
| *Onthophagus marginifer* Frey |  | 1 | 4 |  | 2 | 7 | 27 | 41 |
| *Onthophagus* indet. sp. 11 |  |  |  |  | 2 |  |  | 2 |
| *Onthophagus rufonotatus* d'Orbigny |  |  | 3 |  |  | 1 | 10 | 14 |
| *Onthophagus* sp. n. 8 | 1 | 11 | 17 |  | 1 | 1 |  | 31 |
| *Onthophagus jacksoni* d’Orbigny |  |  | 5 |  |  | 1 | 1 | 7 |
| *Onthophagus* sp. n. 9 |  | 3 |  |  | 4 |  |  | 7 |
| *Onthophagus longipilis* d'Orbigny |  | 1 | 7 |  |  | 1 |  | 9 |
| *Onthophagus* sp. 18 |  |  | 1 |  |  |  |  | 1 |
| *Onthophagus bidentifrons* d'Orbigny |  |  | 2 |  |  |  |  | 2 |
| *Proagoderus elgoni* d'Orbigny |  |  | 7 |  |  | 6 | 1 | 14 |
| *Proagoderus multicornis* d'Orbigny |  | 7 |  |  | 2 |  |  | 9 |
| *Sisyphus* indet. sp. 1 |  |  | 30 |  |  |  | 1 | 31 |
