# Supplementary material for: The Role of Species Traits in Mediating Functional Recovery during Matrix Restoration
Source: PLoS One. 2014 Dec 12;9(12):e115385. doi: 10.1371/journal.pone.0115385 (PMC4264948; doi:10.1371/journal.pone.0115385)
Supplement: S3 Table — Akaike Information Criterion (AIC) scores obtained from the edge function fitting procedure. (DOCX) [file pone.0115385.s007.docx]

**Table S3**. **Akaike Information Criterion (AIC) scores obtained from the edge function fitting procedure.** AIC scores are obtained from models for total dung beetle biomass and dung removal rates at degraded versus regenerating matrix sites. Values are zero-standardized to the AIC scores obtained from the best-fit edge model out of five models of increasing complexity (null, linear, exponential, logistic, and unimodal). A zero denotes the model of best fit and a ‘—’ indicates where the model did not fit the data. Models within ≤ 2 AIC units of the lowest AIC value have equivalent likelihood of being the best model.

| **Response** | **Edge response model** | | | | |
| --- | --- | --- | --- | --- | --- |
|  | **Null** | **Linear** | **Exponential** | **Logistic** | **Unimodal** |
| **Total beetle mass** |  |  |  |  |  |
| Degraded matrix | 6.54 | 0.00 | — | — | 1.14 |
| Regenerating matrix | 22.13 | 0.00 | — | 3.32 | 85.15 |
| **Dung removal** |  |  |  |  |  |
| Degraded matrix | 9.13 | 3.86 | — | 4.40 | 0.00 |
| Regenerating matrix | 20.56 | 0.00 | — | 2.73 | 0.50 |
