# Supplementary material for: The Role of Species Traits in Mediating Functional Recovery during Matrix Restoration
Source: PLoS One. 2014 Dec 12;9(12):e115385. doi: 10.1371/journal.pone.0115385 (PMC4264948; doi:10.1371/journal.pone.0115385)
Supplement: S4 Table — Complete basis set of independence claims for the selected best-fit path model. (DOCX) [file pone.0115385.s008.docx]

**Table S4. Complete basis set of independence claims for the selected best-fit path model.** The ‘Endogenous variable’ column indicates the variable on which the independence claims are being tested. The independence claim being tested is denoted by the last variable in each independence claim model. ‘edge.dist’ = distance from edge, ‘restoration’ = matrix restoration, ‘pronotum’ = pronotum width, ‘BSI’ = body size index, ‘Fdis’ = functional dispersion, ‘t.biomass’ = total beetle mass, and ‘removal’ = dung removal.

| **Endogenous variable** | **Independence claim models** |
| --- | --- |
| Body mass | body mass ~ edge.dist |
|  | body mass ~ restoration |
|  | body mass ~ edge.dist : restoration |
| Pronotum width | pronotum ~ edge.dist : restoration + edge.dist |
|  | pronotum ~ edge.dist : restoration + restoration |
| Wing area | wing area ~ edge.dist |
|  | wing area ~ restoration |
|  | wing area ~ edge.dist : restoration |
| Wing loading | wing loading ~ wing area + pronotum + restoration : edge.dist + body mass |
|  | wing loading ~ wing area + pronotum + restoration : edge.dist + restoration |
| Body size index | BSI ~ edge.dist + wing area + body mass + pronotum + restoration |
|  | BSI ~ edge.dist + wing area + body mass + pronotum + edge.dist : restoration |
| Functional dispersion | Fdis ~ body mass + pronotum + wing area + edge.dist |
|  | Fdis ~ body mass + pronotum + wing area + restoration |
|  | Fdis ~ body mass + pronotum + wing area + edge.dist : restoration |
|  | Fdis ~ body mass + pronotum + wing area + edge.dist + BSI |
|  | Fdis ~ body mass+pronotum+wing area+edge.dist+edge.dist : restoration+wing loading |
| Total beetle mass | t.biomass ~ wing loading + body mass + restoration + edge.dist + protection : distance |
|  | t.biomass ~ wing loading+body mass+restoration+edge.dist+wing area+pronotum+BSI |
|  | t.biomass ~wing loading+body mass+restoration+edge.dist+protection: distance+pronotum |
|  | t.biomass ~ wing loading + body mass + restoration + edge.dist + wing area |
| Dung removal | removal ~ t.biomass + pronotum + restoration : edge.dist + edge.dist |
|  | removal ~ t.biomass + pronotum + restoration : edge.dist + restoration |
|  | removal ~ t.biomass + pronotum + restoration : edge.dist + body mass |
|  | removal ~ t.biomass+pronotum+restoration : edge.dist+edge.dist+wing area+wing loading |
|  | removal ~ t.biomass+pronotum+restoration: edge.dist+edge.dist+wing area+body mass+BSI |
|  | removal ~ t.biomass + pronotum + restoration : edge.dist + wing area |
|  | removal ~ t.biomass + pronotum + restoration : edge.dist + wing area + body mass + Fdis |
