# Supplementary material for: The Role of Species Traits in Mediating Functional Recovery during Matrix Restoration
Source: PLoS One. 2014 Dec 12;9(12):e115385. doi: 10.1371/journal.pone.0115385 (PMC4264948; doi:10.1371/journal.pone.0115385)
Supplement: S1 Appendix — Measuring rates of dung removal. (DOCX) [file pone.0115385.s009.docx]

**Appendix S1. Measuring rates of dung removal**

All dung used for an entire site came from a single homogeneous batch of freshly-mixed pig dung (less than 12 hours old). In order to accurately determine the mass of dung removed by dung beetles, it was essential to know the initial and final moisture contents of the dung (which might vary between batches and also with environmental conditions in the field). Therefore, the 40 gram samples of dung were weighed out from that day’s homogeneous batch and wrapped individually in plastic bags in order to prevent desiccation of the dung (as well as inadvertent colonisation by insects) before placing in the field. At the same time, another fresh 40 g subsample from the same batch was also weighed out and placed directly in a drying oven at approximately 80 °C for at least 48 hours until constant dry mass was achieved, and this value used to determine batch moisture content. The experimental dung samples were left on the surface of the soil for 24 hours and then any remaining dung re-collected, being careful to avoid collecting any debris. In the laboratory, any invertebrates found in the remaining dung were carefully removed to avoid bias in dung mass loss estimates. The dung samples were then put into the drying oven in paper envelopes at approximately 80 °C for a minimum of 48 hours until a constant dry mass was achieved. To calculate the proportion dry mass of dung removed during the 24-hr period we used the formula:

Mass loss = (initial wet mass * (1 – initial batch moisture content)) – final dry mass
