# Supplementary material for: The Role of Species Traits in Mediating Functional Recovery during Matrix Restoration
Source: PLoS One. 2014 Dec 12;9(12):e115385. doi: 10.1371/journal.pone.0115385 (PMC4264948; doi:10.1371/journal.pone.0115385)
Supplement: S2 Appendix — Measurement of dung beetle traits. (DOCX) [file pone.0115385.s010.docx]

**Appendix S2. Measurement of dung beetle traits**

In order to facilitate trait measurements, individuals from each species in each pitfall sample were pinned using standard stainless steel (#2) entomological pins. All pins were individually pre-weighed for each beetle specimen to the nearest 0.001 mg using a Mettler Toledo UMX2 ultrafine microbalance. The absolute range of variation in pin mass was 4.029 mg (45.970 – 49.999 mg), which far exceeded the mass of the smallest beetle specimen (0.227 mg). For any given trap sample, all individuals were pinned for species that were represented by less than 20 individuals (in that sample), but for logistical reasons this was not possible for very abundant species that had more than 20 individuals (in that sample). For these abundant species, a random subsampling procedure was employed to reduce the number of individuals pinned. We placed all individuals of the abundant species into a Petri dish marked with eight equal radiating segments, and spread them approximately evenly across the dish. Each segment was assigned a number from one to eight and a random number generator was used to pick a segment from which to subsample beetles. All individuals in that segment were pinned and measured. If the total number of beetles from the first randomly-drawn segment was less than 20, all beetles in the next segment in a clockwise direction were also pinned and measured, and so on until more than 20 individuals were obtained. The nature of the subsampling procedure meant that often substantially more than 20 beetles were pinned from some very abundant species in some trap samples. We excluded one species, *Diastellopalpus tridens*, which was only represented by three individuals in total and all were damaged, thus traits could not be measured.

Immediately after pinning, when specimens were still moist and flexible, the left hind wing of each specimen was removed using fine forceps for morphometric analysis. The wing was then mounted and spread open on a microscope slide using glycerine jelly with 2 % phenol as a preserving agent. This mounting agent solidifies at room temperature, therefore the slide was kept warm on a heating element while the wing was being mounted, and was then cooled immediately after a coverslip had been placed over the wing. Subsequently, a digital image was taken of each wing using a Nikon D40 SLR camera with a macro lens, mounted at a fixed height directly above the slide. A 1 cm scale bar was placed next to the slide-mounted beetle wing in order to calibrate wing measurements.

Digital images were processed using Adobe Photoshop CS2 in order to obtain a measure of wing area. This was achieved by digitally clipping out the wing from the image background and then digitally filling in the wing area with black on a white background. In order to standardise the point at which each wing was cut at the base, all images were clipped in a plane bisecting homologous vein junctions (Figure S3). The image file was then saved as a bitmap and imported into Image J software to calculate total image area from the scale bar, and proportion of total area represented by black pixels. This gave wing area in mm^2^ for one wing which was then multiplied by two for an approximation of absolute wing area per beetle.

Body size was measured using the width of the pronotum as a simple linear surrogate of overall size. This measure was the most suitable as other measures such as body length can vary idiosyncratically in beetles due to the expansion or contraction of the soft tissues at arthrodial joints. Furthermore, pronotum width in dung beetles has been found to be positively correlated with female reproductive output and mating success in males [1-3] and is therefore a useful indirect measure of beetle fitness.

Once individuals were pinned, and the left wing removed, they were stored in a cool dark room for a minimum of four months to air-dry. Specimens smaller than 300 mg were put in a drying oven at 60 °C for two hours to achieve complete drying and immediately weighed to the nearest 0.001 mg as described above. The required drying time was established by repeatedly re-weighing a test set of beetles in the drying oven until the specimens reached a constant mass. For all specimens larger than 300 mg, individual drying times were continued beyond two hours at 60° C until each specimen reached a constant mass. The mass of each beetle was then calculated by subtracting the pre-recorded mass of that individual pin from the total mass of the beetle plus pin after drying. For trap samples in which some individuals of abundant species were not pinned (i.e. were not in the subsample selected for pinning and trait measurements), we randomly selected values within species for each trait by assigning each value a number ascending from one to the last value and then using a random number generator to select associated trait values. We then applied these trait values to all remaining individuals of the respective species from that sample. This ensured that real levels of variation in traits were maintained within species collected in trap samples.

**References**

1. Kotiaho Janne S, Simmons Leigh W, Hunt J, Tomkins Joseph L (2003) Males influence maternal effects that promote sexual selection: a quantitative genetic experiment with dung beetles *Onthophagus taurus*. The American Naturalist 161: 852-859.

2. Hunt J, Simmons LW (2001) Status-dependent selection in the dimorphic beetle *Onthophagus taurus*. Proceedings of the Royal Society of London Series B-Biological Sciences 268: 2409-2414.

3. McLain DK (1991) Heritability of size: a positive correlate of multiple fitness components in the southern green stink bug (Hemiptera, Pentatomidae) Annals of the Entomological Society of America 84: 174-178.
