# Supplementary material for: The Role of Species Traits in Mediating Functional Recovery during Matrix Restoration
Source: PLoS One. 2014 Dec 12;9(12):e115385. doi: 10.1371/journal.pone.0115385 (PMC4264948; doi:10.1371/journal.pone.0115385)
Supplement: S3 Appendix — Continuous edge response models. (DOCX) [file pone.0115385.s011.docx]

Appendix S3. Continuous edge response models

Using a form of the general logistic model we determined the best-fit edge model out of five models of increasing complexity [1]:

(1) the null hypothesis of no discernible edge effect, calculated as the mean of the response variable η:

$$\eta=\overline{\eta}+\varepsilon$$

where ε is an error term;

(2) a simple linear gradient of the form:

$$\eta_{D}=\beta_{0}+\beta_{1}D+\varepsilon$$

where β_0_ and β_1_ are constants and *D* is the distance to edge;

(3) a power model:

$$\eta_{D}=\beta_{0}+\beta_{1}e^{\beta_{2}D}+\varepsilon$$

(4) a logistic model that describes a sigmoidal change in community composition across an edge, with an asymptote in both the patch and matrix habitats:

$$\eta_{D}=\beta_{0}+\frac{\beta_{1}-\beta_{0}}{1+e^{(\beta_{2}-D)\beta_{3}}}+\varepsilon$$

which also includes an additional constant, β_3_;

(5) a unimodal model based on the logistic model, but with one extra constant (β_4_) and a *D*^2^ term to describe a unimodal change in community composition at a particular distance from an edge:

$$\eta_{D}=\beta_{0}+\frac{\beta_{1}-\beta_{0}}{1+e^{(\beta_{2}-D+\beta_{4}D^{2})\beta_{3}}}+\varepsilon$$

We fitted these five models to forest-to-matrix edge gradients, treating the three edge gradients within each of the two matrix restoration treatments (degraded versus regenerating matrix) as replicates. In each case, we assessed model significance and calculated the Akaike Information Criterion (AIC) value for each model. We selected the best model as the one with the lowest AIC value, or in the case of multiple models within two AIC units of each other, we selected the simplest model (with the fewest parameters). Model fitting was conducted in R version 2.5.1 (R Development Core Team 2004).

**References**

1. Ewers RM, Didham RK (2006) Continuous response functions for quantifying the strength of edge effects. Journal of Applied Ecology 43: 527-536.
