# Supplementary material for: The Role of Species Traits in Mediating Functional Recovery during Matrix Restoration
Source: PLoS One. 2014 Dec 12;9(12):e115385. doi: 10.1371/journal.pone.0115385 (PMC4264948; doi:10.1371/journal.pone.0115385)
Supplement: S4 Appendix — Constructing generalised multilevel path models. (DOCX) [file pone.0115385.s012.docx]

**Appendix S4. Constructing generalised multilevel path models**

Due to the hierarchical nature of the data, we used generalised multilevel path models which are also highly flexible in dealing with interactions [1]. To test the validity of a multilevel causal path model, several steps must be taken. Firstly, we identified the 'basis set' ***B****_U_* of independence claims that are implied in a directed acyclic causal diagram (i.e. a unidirectional box-and-arrow diagram). ***B****_U_* expresses the full set of independence claims (i.e. pairs of variables in the acyclic model with no arrow between them) and dependence claims (pairs of variables in the model with a causal arrow joining them). Secondly, we determined the probabilities *p_i_* for each of the *k* independence claims in ***B****_U_* using linear mixed effects models for normally-distributed response variables and generalised mixed effects models for binomially-distributed response variables. The combined *p_i_* of the full model was calculated as $C= -2\sum_{i=1}^{k} ln(p_{i})$, and the *C* statistic was then compared to a chi-square (*χ*^2^) distribution with 2*k* degrees of freedom [1]. This gives the probability *P* that the model does not depart significantly from what would be expected under such a causal model [1]. A model can be rejected if the *P*-value derived from the *C* statistic is smaller than the specified α-level (in this case α = 0.05). Therefore, if *P* > 0.05 the causal model is not rejected and provides a good fit to the data.

Linear mixed effects models were fitted using the ‘nlme’ package in R 3.0.1 [2], with ‘transect’ (i.e. the three replicates of forest-to-matrix edge gradients in degraded versus regenerating matrix sites) specified as a random factor for all tests. Assumptions of normality were tested for linear models by inspecting the variance structure of each model. Dung beetle body mass was log-transformed and all predictors in the model were mean-centred by subtracting the mean of a given variable from each value of that variable.

To fit the individual path coefficients that lead to endogenous variables (measured variables within the model that have arrows leading to them) we used restricted maximum likelihood (REML) estimation and tested for their significance. As all predictors in the model were mean-centered, unstandardised path coefficients could be interpreted as the degree of change in the response variable for a given unit change in the predictor. However, interaction terms are interpreted differently whereby the coefficient indicates the amount of change in the slope of the regression of the response variable against a predictor following a unit change in the other interacting predictor variable [3].

Model fit was assessed using the procedure for calculating R^2^ values for generalized linear mixed effects models as outlined by Nakagawa and Shielzeth 2013 [4]. This provides a clear measure for the proportion of total variation of endogenous variables explained by all significant predictors. Standardised path coefficients were calculated to assess relative predictive strength of each predictor on endogenous variables. This was done by taking the mean-centered values of each predictor and dividing each value by twice the standard deviation of the respective predictor, as recommended by Gelman 2008 [5]. After re-running the model with standardised variables, standardised path coefficients were interpreted in a similar way to the unstandardised coefficients, except that units of change are expressed in units of standard deviation. Therefore, standardised path coefficients can be directly compared between effects and thus indicate the relative importance of each path.

**References**

1. Shipley B (2009) Confirmatory path analysis in a generalized multilevel context. Ecology 90: 363-368.

2. R Development Core Team (2013) A Language and Environment for Statistical Computing. Vienna, Austria: R Foundation for Statistical Computing.

3. Aiken LS, West SG (1991) Multiple regression: testing and interpreting interactions. Thousand Oaks, USA: Sage Publications.

4. Nakagawa S, Schielzeth H (2013) A general and simple method for obtaining R2 from generalized linear mixed-effects models. Methods in Ecology and Evolution 4: 133-142.

5. Gelman A (2008) Scaling regression inputs by dividing by two standard deviations. Statistics in Medicine 27: 2865-2873.
